# Editorial: Biophilic design rationale: Theory, methods, and applications

**DOI:** 10.3389/fpsyg.2022.978689

**Published:** 2022-10-06

**Authors:** Rita Berto, Giuseppe Barbiero, Jack L. Nasar

**Affiliations:** ^1^GREEN LEAF - Groupe de Recherche en Education à l'Environnement et à la Nature, Laboratorio di Ecologia Affettiva, Université de la Vallée d'Aoste, Aosta, Italy; ^2^City and Regional Planning, Knowlton School of Architecture, The Ohio State University, Columbus, OH, United States

**Keywords:** biophilia hypothesis, restorative environments, architecture, design, methods

“Humanity gradually realizes that what is common to man is more important than what is different...” Thornton Wilder

Architecture cannot be limited to objects evaluated through the history of art, because what humans like, in a broader sense, does not necessarily relate just to aesthetics (appearances) but to what they biologically need. Considering architecture in these latter terms leads to quite different insights into the meaning of buildings and human dwellings. Barbiero and Berto's review places evolution as central to understanding modern human relations with the environment, outlining that aesthetic appraisal evolved also to support our informational needs (making sense, exploring solutions for adaptation), steering people toward psychological benefits (e.g., stress recovery and attentional restoration). In this regard, Robles et al.'s study reaffirms the universal impact of fractal patterns on viewers and demonstrates how fractal design balances aesthetic and psychological needs while serving as a practical implementation of biophilic patterns in human-made environments to promote occupant wellbeing. This means that architecture is a human phenomenon that extends over individuals and cultures with origins from a biological need called *biophilia* and evidence-based psycho-physiological restorative effects. Biophilic design can rely on a robust evolutionary theoretical framework, but because most of this research is non-experimental, it has not shown correlation or causality. This may be related to inductive and deductive research approaches, which in turn fuel confusion between restorative design and biophilic design. Studies adopting the inductive approach are essentially based on perception, observation, and measurement of phenomena from which they attempt to derive generalizations and scientific assumptions, which are verified according to well-defined terms. Studies of this type tend to center on restorative environment theories (Attention Restoration Theory, Stress Recovery Theory), as Neilson et al.'s mini review discusses. Since astronauts living in space will be unable to access natural landscapes, which have been found to have restorative effects on stress and wellbeing, the authors suggest providing a restorative environment design as a relatively simple and cost-effective way to mitigate the stress faced during long-duration space missions. It is no coincidence that many 3D reconstructions of the holodeck from the starship in the *Star Trek* television series have Nature as their background. Martinez-Soto et al. related affective responses to the properties of 65 public spaces in a Mexican city. The study found positive effects on mood and reduced stress related to the restorative properties of the spaces. Boffi et al. used focus groups with older people to co-design a community garden. The study found that the needs expressed by the groups fit the Attention Restoration Theory components of compatibility, fascination, and being away. Studies with a deductive approach, in contrast, begin with generally valid assumptions of biophilia and the biophilia hypothesis and proceed to the interpretation of the individual case from the theoretical top, down to the base. Gaekwad et al.'s meta-analysis provides evidence for fundamental theories regarding the human-Nature biophilic bond, while not ascertaining clear support for the biophilia hypothesis due to the broader definition of the hypothesis itself and the lack of studies of human response pathways associated with the biophilia hypothesis. Starting from the biophilic theory, Pasini et al. created, in a shared design process with worker representatives, a new workplace. However, despite the opposite approach and proximity to restorative theories or biophilia hypothesis, all studies agree that human nature and human relationships with Nature should be central to design. Our relationships with Nature have changed over the course of our evolution. Our biophilia evolved in the Paleolithic era as an evolutionary adaptation, to favor the recognition of resources and suitable refuges in the wilderness. The Neolithic revolution redefined our relationship with both resources and refuges. Plants cultivation and animal breeding required the abandonment of nomadic life, and the construction of stable refuges that were able to protect resources. If we accept that spatial structure was generated by these evolutionary needs and these needs influenced humans along the anthropological continuum, then we could theorize the “object” architecture on the base of what is biologically common to human beings. Biophilic design can provide a new, previously unknown paths in this direction.

## Author contributions

All authors listed have made a substantial, direct, and intellectual contribution to the work and approved it for publication.

## Conflict of interest

The authors declare that the research was conducted in the absence of any commercial or financial relationships that could be construed as a potential conflict of interest.

## Publisher's note

All claims expressed in this article are solely those of the authors and do not necessarily represent those of their affiliated organizations, or those of the publisher, the editors and the reviewers. Any product that may be evaluated in this article, or claim that may be made by its manufacturer, is not guaranteed or endorsed by the publisher.

